# Foreign Correspondence

**Published:** 1850-11

**Authors:** 


### FOREIGN CORRESPONDENCE.

The excellent foreign correspondent of this *Journal*, in a letter addressed to one of the editors, under date of October 1st, announces that the medical teaching of Paris was at that time enjoying a vacation, and that he had but little of interest to communicate. The following statements contained in his letter, have novelty and importance enough to entitle them to a more prominent place than in a private correspondence and we present them to the readers of the Journal of Medicine:

"Physical signs of diseases of the chest are undergoing important changes. 1st. It is *said* that false membrane gives rise to dullness on percussion. (?) 2nd. In pleurisy, with effusion and adhesion binding the lungs to the costal pleura, we have a *tympanitic* sound on percussion, proved by percussing a lung floating upon the surface of water. 3rd. Super-inflation, if I may use the term, that is forced distension *by air*, gives rise to a DULL sound on percussion, proved by forcibly distending the lungs with air, and percussing thereon.

You may recollect that some time since, M. Piorry made a great deal of noise about the almost immediate diminution of the volume of the spleen, in intermitting fever, upon the exhibition of the sulphate of quinine, which, by-the-bye, he always gave in solution. This is not only true, but the vehicle without the quinine, or simple water, and I've seen it tried, produces the same result. In intermitting fever, you know the spleen is always more or less enlarged; find one of these cases, and by percussion, properly employed, it is possible to determine very accurately the limits of the organ; give the patient a glass of water, and in a few minutes, not exceeding three, the volume of the spleen is found, upon again percussing, to be sensibly diminished. If this proves anything, does it not prove that the spleen is a reservoir of blood for the stomach during its quiescent state?

"Mr. Berrard, a physiologist of talent and promise, demonstrates, among many other curious things, that the temperature of the blood in the vena cava, after its exit from the liver, is 1½ degrees higher than it is in the vena portarum, before ramifying through that organ; and furthermore, that it loses this 1½ degrees whilst circulating in the pulmonic capillaries. That there is no sugar in the portal vein before it enters, whilst it abounds in the cava after it leaves the liver: that there is a direct communication between the hepatic vein and the vena cava: that there is a direct communication by regurgitation, between the inferior cava and the kidneys; and this he proves by giving strychnine to rabits, etc., and after the lapse of some minutes (15) innoculating the urine in the neck, and causing. by the innoculation, all the accidents usually resulting from poisoning by strychnine. He is now engaged in writing a work on physiology, destined, I think, to change the whole science of physiology.

"If you are already informed on these various changes, you will doubtless find their repetition here a great bore; but if they are new to you, their novelty and interest will fully apologise for the manner in which they are told. Believe me, dear doctor,

"Your friend and servant, "R. P. H."

